# Annotations

**Published:** 1893-08-12

**Authors:** 


### Annotations.

**Do we Starve Our Children?**

In an American paper there appeared a little time ago a comparison between the way children were fed in England and the United States. From that it certainly appears that, give the average schoolboy his choice, and he would not, like the patriotic sailor of Mr. Gilbert's vision, elect to remain an Englishman. The American child breakfasts off rich and highly spiced dishes, has stewed oysters and ice-cream *ad libitum* for lunch, and enjoys a dinner that is like a Christmas dream every day. The writer declares that there is not a moment in the life of an English child when he is not hungry, that he will not complain at being disturbed in his first sleep if the awakening is compensated by bread and jam; that never, through all the eager years of growth, does he know the sensation of satisfied appetite. Perhaps this is an over-statement, but certainly, from the trans-atlantic point of view, the British routine of bread and milk or oatmeal porridge, roast mutton and rice pudding, probably seems little short of starvation. But what is the result? The Englishman has at least a fair hygienic start in life; with his most conscientious efforts in self-indulgence he does not greatly suffer from indigestion before he is forty, while the average American is a dyspeptic from his youth up. Climate may to some extent be responsible for this, the strain and stress of life in a country where it is the bounden duty of every citizen to become a millionaire or perish in the attempt is in some degree to blame, but who can doubt that this early over-feeding is at the root of all. Not that we recommend the starvation of children; on the contrary, we think that some parents are a little too anxious to guard against the dangers of repletion, and that some well-meant books err in fixing the exact number of ounces a child should eat at a given age. The average child has a much more accurate indicator within himself, and while the food set before him is plain and wholesome, not of a nature to artificially excite the appetite, the internal test may safely be trusted. It is only when the taste for bread and butter vanishes while the taste for cake remains in full force, that interference is necessary, and to keep a child sound and strong abundance of exercise is better than scarcity of food. Here our American cousins misjudge us. We do not, as they fancy, starve our children, but we insist on their working off their food by physical activity. On the food question we look beyond the palate. Breakfast and dinner are not mere gratifications, they are potential bone and muscle, and let the pampered American tantalise us as he will with terrapin and ice-cream and candy, we will continue to restrict our children's diet to the things which make bone and muscle of the best.

**The Dangers of "Safe" Hypnotics.**

There are some medical men who prescribe hypnotics with a very light heart; there are others who never prescribe them at all, except under the sternest compulsion. Among the latter class Sir Andrew Clark may, we believe, be included. In our judgment, Sir Andrew Clark and those who agree with his practice in this matter are entirely right; whilst the too ready prescribers of hypnotics, even of the "safest" class, deserve the severest professional reprehension. Dr. Frank Ashby Elkins, Senior Assistant Physician to the Royal Edinburgh Asylum, has just published an account of the case of a man who has experienced extraordinary sufferings as the result of the "paraldehyde habit." The victim, in addition to his profound miseries, was for a long time within sight of death's door. The case is published as a warning both to doctors and patients. The patient, A. B., was a steady, respectable coachman sixty-five years of age, and last November, he, in desperation, voluntarily sought admission into the Royal Edinburgh Asylum, in order, if possible, to be cured of the habit which, besides driving him to desperation, was killing him by inches. For seven years A. B. had been subject to insomnia. Under medical advice, and in a fatal moment, he tried paraldehyde. Beginning with occasional small doses, the unfortunate man rapidly proceeded to frequent large ones. In a few months he could not live without the drug; and in a very few years it became painfully evident that he could not live with it. Life became an agony, and the apprehension of death a terror which was intolerable. Physically he became pale and emaciated, losing two stones in weight in six months. So weak was he that he was confined to bed, and could not even lift his food to his mouth. His wife had to feed him with a spoon like a child. In course of time the drug lost its power, and sometimes as much as seven teaspoonfuls of paraldehyde procured no more than half an hour's sleep. The heart's action failed, and the sounds often became almost inaudible. Peculiar shivers ran through the body. "Strange beasts," the patient declared, paced about his room; and he felt certain that the doctors and nurses were leagued together to poison him. He constantly walked up and down his room, by night as well as by day, in terror lest he should die if he lay down in bed. He was certain the house was on fire. Tremors, agitations, suspicions, delusions, harassed him day and night, until finally he lost his reason, and was certified as a lunatic requiring restraint. Such is the story. After a few months of asylum life recovery commenced, and in time the man was released and returned to work. But his constitution is permanently impaired, and he will never again be a sound and healthy man. The case is one which should impress upon every medical practitioner and every patient that hypnotics of every class are dangerous remedies, and must never be resorted to except occasionally and under the compulsion of the most absolute necessity.

**The Sick-room.**

When laid low by illness, it not infrequently becomes patent to the sufferer that the equipments of the bed-room do not lend themselves to a patient's wellbeing. A patient knows very well when he is not at ease, but he is not in the condition to explain what is wrong and just what he wants, and so, may be, the light—theoretically so excellent in illness—streams on to weary eyes, because it never dawns on an attendant spirits to move the bed, or place head to foot. A candle remains unshaded, one ray of light dances on the wall in the early morning through partly closed curtains; the creaking door handle is never oiled; a loose board is constantly chosen as a favourite pathway, and a thousand other little jars to the patient's nerves are entirely ignored. Last, but not least, remains the frenzy of the wall paper. This cannot be altered by our sympathisers, even when they would, and so this is a case essentially for prevention not cure. There is an idea prevalent in many minds that bed-room papers "don't matter." Any horrid, worrying, little spotty pattern that is cheap will do for the bed-room, until we are ill, and then the ignored revenges itself. It falls but to the lot of the favoured few never to have occupied their bed-room but in health. This should be borne in mind, not overlooked, as it always is, when bed-rooms are equipped. Something unobjectionable to nerves in sickness might easily be chosen if only a little thought were bestowed. Not only an absence of an irritating pattern, but the presence of a pleasant tone of colour is desirable. Bilious greens or gloomy mixtures are neither good in sickness nor in health. We may not, perhaps, in health be so conscious of their effect on our spirits, but an effect they have nevertheless. Ill or well, we should create our sunshine from within, and thus render the tedious period of sickness at any rate a little more bearable.

